# Physical health-related quality of life trajectories over two years following breast cancer diagnosis in older women: a secondary analysis

**DOI:** 10.1007/s00520-024-08475-6

**Published:** 2024-04-11

**Authors:** Shan S. Wong, Beverly J. Levine, Kimberly J. Van Zee, Elizabeth Z. Naftalis, Nancy E. Avis

**Affiliations:** 1 Present Address: Department of Mental Health & Behavioral Sciences, West Palm Beach Veteran Affairs Healthcare System, 7305 N Military Trl, West Palm Beach, FL 33410 USA; 2 Department of Social Sciences and Health Policy, Wake Forest University School of Medicine, Medical Center Blvd, Winston-Salem, NC 27157 USA; 3 Department of Surgery, Breast Service, Memorial Sloan Kettering Cancer Center, 300 East 66th Street, New York, NY 10065 USA; 4 Department of General Surgery, Baylor University Medical Center, 4001 Worth St, Dallas, TX 75246 USA

**Keywords:** Breast cancer, Comorbidities, Older, Physical Health, Quality of Life, Trajectories

## Abstract

**Purpose:**

To identify distinct trajectories of physical health-related quality of life (HRQoL) in older women over the first two years following breast cancer diagnosis, and to examine characteristics associated with trajectory group membership.

**Methods:**

A secondary analysis of a longitudinal study of women diagnosed with stage I-III breast cancer who completed surveys within eight months of diagnosis and six, twelve, and eighteen months later that focuses on a subset of women aged ≥ 65 years (*N* = 145).Physical HRQoL was assessed using the Physical Component Score (PCS) of the SF-36 Health Survey. Finite mixture modeling identified distinct PCS trajectories. Multivariable logistic regression identified variables predictive of low PCS group membership.

**Results:**

Two distinct patterns of PCS trajectories were identified. The majority (58%) of women had PCS above the age-based SF-36 population norms and improved slightly over time. However, 42% of women had low PCS that remained low over time. In multivariable analyses, older age, difficulty paying for basics, greater number of medical comorbidities, and higher body mass index were associated with low PCS group membership. Cancer treatment and psychosocial variables were not significantly associated.

**Conclusion:**

A large subgroup of older women reported very low PCS that did not improve over time. Older age, obesity, multiple comorbidities, and lower socioeconomic status may be risk factors for poorer PCS in women with breast cancer. Incorporating routine comprehensive geriatric assessments that screen for these factors may help providers identify older women at risk for poorer physical HRQoL post breast cancer treatment.

## Introduction

The number of women diagnosed with breast cancer aged ≥ 65 years in the U.S. is rising substantially, largely due to the aging population [1]. By 2030, the estimated number of new cases of invasive breast cancer diagnosed in women aged ≥ 65 years in the U.S. is projected to be 179,000, representing a 57% increase from 2010 [2]. Given this demographic increase, greater attention has been directed at the impact of cancer on the health-related quality of life (HRQoL) in older cancer survivors [3–5].

HRQoL refers to the impact of health on the physical, emotional, and social domains of life [6]. In general, older age is associated with poorer physical HRQoL [7, 8], which may be attributed to a greater number of medical comorbidities that can impact health, physical functioning, and survival [9]. Older cancer survivors are even more vulnerable to poorer physical HRQoL compared to age-matched adults without a history of cancer [10, 11]. Population-based case–control studies suggest that women with breast cancer aged ≥ 65 years report significantly poorer physical HRQoL compared to their matched counterparts without a history of cancer, particularly in the early years following breast cancer treatment [11–13].

Despite a growing body of literature on the HRQoL of older women with breast cancer [12, 14–24], the majority of these studies examine older patients as one age group. However, individuals aged ≥ 65 years comprise a highly heterogeneous group, varying in both physical health status and functioning [25–27]. Examination of this heterogeneity may help to identify characteristics of survivors who have different patterns in physical HRQoL trajectories and, consequently, identify those patients most at risk. To date, four studies have examined the physical HRQoL trajectories of breast cancer survivors [28–31]. None of these studies, however, focused on women age ≥ 65.

The present study extends the literature by exploring physical HRQoL trajectories with a specific focus on older women diagnosed with breast cancer. We include variables that others have found to be related to heterogeneity in HRQoL such as breast cancer treatment, medical comorbidities, and psychosocial variables [28, 30, 32]. Our objectives were: (1) to determine physical HRQoL trajectories among breast cancer survivors aged ≥ 65 years; and (2) to identify sociodemographic, cancer-related, health-related, and psychosocial factors associated with trajectory group membership.

## Materials and methods

### Study population and procedures

This is a secondary analysis of a longitudinal study of women aged 18 and older following a first-time diagnosis of breast cancer [33]. The present analyses focus on physical HRQoL of women diagnosed at age ≥ 65 years.

Details on study design has been previously described [33]. Briefly, patients were recruited from Memorial Sloan Kettering Cancer Center (MSKCC) and the University of Texas Southwestern Center for Breast Care in 2002–2006 and followed until 2008. Eligibility criteria included a first-time diagnosis of stage I-III breast cancer and ability to read and write in English. Baseline questionnaires were completed by mail within eight months of diagnosis. Follow-up questionnaires were administered at six, twelve, and eighteen months after baseline. All questionnaires were returned to the coordinating center at Wake Forest University School of Medicine. Date of diagnosis and date of survey completion were used to create a continuous variable of time since diagnosis (in months). Thus, longitudinal data collected ranged from 0 months (three days) to 26 months post diagnosis. All sites had approval from their Institutional Review Boards and met the requirements for the protection of human subjects (Wake Forest School of Medicine protocol #BG01-100, MSKCC protocol #01-120A, and UT Southwestern protocol #0501–260). Informed consent was provided by all study participants.

### Measures

*Primary Outcome.* Physical HRQoL was measured by the Physical Component Summary (PCS) from the Medical Health Outcomes Study Short Form-36 item version (SF-36) [34]. The SF-36 is a widely used measure among all ages and populations and is appropriate for this age group [35–37]. The SF-36 encompasses eight weighted health concepts: physical functioning, role limitations caused by physical health problems, energy/fatigue, pain, general health perceptions, social functioning, emotional well-being, and role limitations caused by emotional problems. Scores on PCS are normed to a T-score metric ranging from 0 to 100, with higher scores reflecting better functioning. U.S. population reference norms [7] on the PCS from the SF-36 User’s Manual was used to compare scores from our sample to women aged ≥ 65 years without a history of cancer.

*Sociodemographic factors* obtained at baseline include: age at diagnosis (continuous), race (white/non-white), college graduate (yes/no), married/partnered (yes/no), and difficulty to pay for basics (e.g. food, housing, medical care, and heating; not very hard/somewhat or very hard).

*Cancer-related characteristics* obtained from chart reviews included: cancer stage (I-III) at diagnosis, mastectomy (versus lumpectomy only), time since diagnosis (months), and receipt of radiation therapy (yes/no) and chemotherapy (yes/no).

*Health-related factors* included body mass index (BMI) and self-reported medical comorbidities obtained at baseline: amyotrophic lateral sclerosis, angina, arthritis, asthma, diabetes, diverticulitis, emphysema, gallbladder disease, glaucoma, heart disease, hypercalcemia, high cholesterol, hypertension, kidney stones, kidney failure, migraine headache, multiple sclerosis, overactive thyroid, underactive thyroid, pancreatitis, ulcer, lupus, and Crohn’s disease. The number of comorbidities was categorized as 0, 1, 2, ≥ 3.

*Psychosocial variables* were collected at multiple survey points. The present analyses used least-squares values of these variables (modeling described below) estimated at 4 months post-diagnosis.

The Illness Intrusiveness Rating Scale (IIRS) [38] assesses the degree to which cancer diagnosis/treatment has intruded on eleven areas of life (we removed health and active recreation because these two items are directly related to our outcome on physical HRQoL). Scores range from 11 to 63, with higher scores indicating greater impacts to life areas.

Coping strategies were measured using the Brief COPE scale [39], a 28-item scale that measures fourteen types of coping strategies. Higher-order exploratory factor analyses of our data revealed two domains of coping: active coping (active coping, emotional support, instrumental support, and positive reframing) and passive coping (self-blame, denial, behavioral disengagement) with scores ranging from 1–4 on each domain [40]. Higher scores reflect higher levels of using that domain.

The RAND Social Support Scale [41] was used to measure social support. This nineteen-item scale measures a person’s evaluation of the functions and resources provided by their social network. Scores range from 19 to 95, with higher scores indicating greater support.

Spirituality was assessed using the Functional Assessment of Chronic Illness Therapy – Spiritual Well-Being Scale (FACIT-Sp) [42]. This thirteen-item scale has two subscales: meaning and peace (possible range: 0–32) and the role of faith in one’s life (possible range: 0–16); higher scores reflect greater spiritual well-being.

Optimism was assessed using the eight-item Life Orientation Test [43]. Scores range from 0 to 32, with higher scores reflecting greater levels of optimism.

### Statistical analyses

SAS version 9.4 of the SAS system for Windows (Copyright (c) 2016 SAS Institute, Inc., Cary, NC. USA) was used for all analyses. To address the first objective, we used group-based trajectory group modeling to identify homogeneous groups with distinct trajectories of PCS as a function of time (months) since diagnosis. We used a combination of statistical criterion (the Bayesian Information Criterion, or BIC) as well as subjective judgment (minimum group size of at least 10% and/or visually distinctively different trajectories) to select the final optimal number of trajectory groups from models that allowed from 2–5 groups. The procedure (PROC TRAJ in SAS) assumes that missing data are missing completely at random [44]. The procedure assigns a posterior probability of group membership for all participants; participants are assigned to the trajectory group for which they have the maximum posterior probability. Trajectories were modeled as a function of time since diagnosis, and both linear and quadratic terms for time since diagnosis were initially included in all models. Observed and predicted means at each time point for the trajectory groups were also reviewed.

To address our second objective, we examined associations between sociodemographic, cancer-related, health-related, and psychosocial variables previously described and trajectory group membership, using chi-square tests (and Fisher’s exact tests, when cell sizes were small) for categorical variables and F tests for continuous variables. For continuous variables that were collected at multiple time points and thus were time-varying (illness intrusiveness, active coping, passive coping, social support, spirituality meaning and peace, spirituality role of faith, and optimism), we estimated values of those variables at a specific post-diagnosis time point (four months post diagnosis) using least-squares estimates from mixed (repeated measures) models containing time in months since diagnosis, time in months squared, trajectory group, and the full interaction (i.e., 2 terms) between trajectory group membership and time since diagnosis. In all models, we employed the unstructured covariance matrix option, to impose as few assumptions as possible within the models. We then tested differences in the mean estimated four-month-post-diagnosis values between trajectory groups using contrast statements within each model. Such modeling enabled us to use a common critical referent (i.e. time at diagnosis) for participants for these variables, rather than using the time axis of survey administration, because participants completed the first survey at varying lengths of time following diagnosis. For other variables in our analyses (i.e., those that either were not collected at all time points or that are intrinsically non-time-varying, such as education), we compared the values from the initial survey.

Variables that differed significantly between trajectory groups at p ≤ 0.1 were entered in a multivariable logistic regression model to examine characteristics associated with the lowest PCS group membership in a multivariable setting.

## Results

### Participant characteristics

Participants were predominantly Non-Hispanic Caucasian (94%) and married/partnered (64%; Table 1). About half were college educated (47%) and few reported difficulty paying for basics (12%). The mean age of the analytic sample was 72.5 years (SD = 6.1, range = 65.1–96.8 years). At baseline, mean time since diagnosis was 4.7 months (SD = 1.1, range 0.2–6.7 months). Most women had stage I breast cancer at diagnosis (65%). Only 6% had stage III breast cancer so we combined those with stage II and III together in analyses. Of the 145 women in the analytic sample, 37% were in treatment at the time of the baseline survey. Most received radiation at some point during the course of their treatment (70%), 38% received chemotherapy, and 81% received hormonal therapy.

**Table 1:** Baseline characteristics of study sample (*N* = 145)

Characteristic	n (%)
*Sociodemographic Variables*	
Age in years at diagnosis, Mean (SD)	72.5 (6.1)
Non-Hispanic Caucasian	137 (94)
College graduate	68 (47)
Married/partnered	93 (64)
Difficulty paying for basics, somewhat/very hard	17 (12)
*Cancer-related variables*	
Cancer Stage	
I	94 (65)
II	42 (29)
III	9 (6)
Mastectomy, yes^a^	36 (25)
Radiation, yes^a^	102 (70)
Chemotherapy, yes^a^	55 (38)
Hormonal therapy, yes^a^	117 (81)
Months since diagnosis, Mean (SD)	4.7 (1.1)
*Health-related Variables*	
BMI, kg/m^2^, Mean (SD)	26.6 (5.9)
Comorbidities, No	
0	14 (10)
1	36 (25)
2	37 (26)
3 +	57 (40)

*Abbreviations:* BMI, Body Mass Index

^a^ at any time following diagnosis

### Description of PCS trajectories

A two-trajectory model produced the largest BIC and was therefore selected. The quadratic terms for time since diagnosis for each trajectory were not significant in the initial model (*p* >  = 0.7 in both cases), and we therefore employed a simpler linear fit only.

**Fig. 1:**
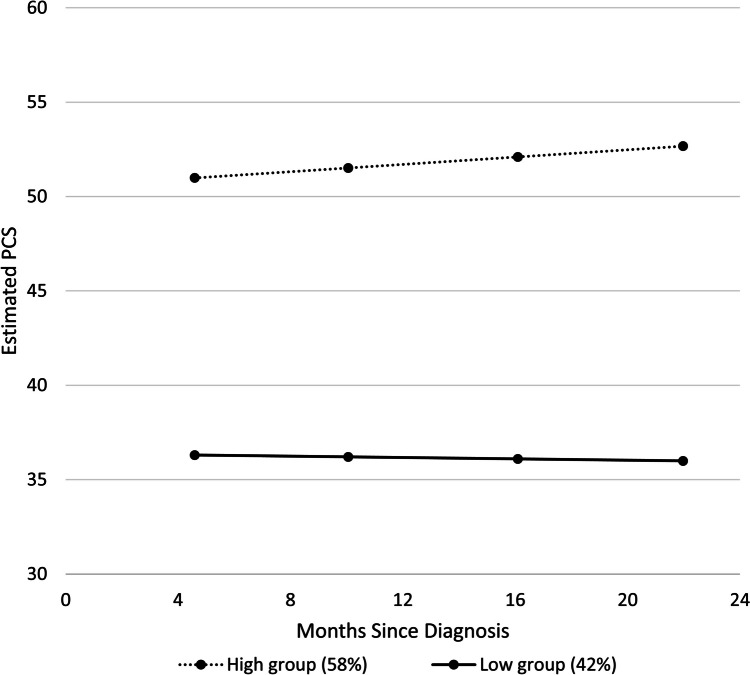
Predicted PCS for each trajectory group by months since diagnosis, with percentage of participants in each group

Two distinct predicted trajectories of PCS emerged from our analyses (Fig. 1). (Note that we plot only the predicted means over time for the two groups, as the observed means were so close to the predicted that they were not distinctly visible in the Figure). The majority (58%) of women were in the High Group with an estimated mean PCS of 50.5 shortly after diagnosis, which is above U.S. population norms of older women without a history of cancer (PCS of 42.2–45.6) [7, 8]. This High Group showed an improvement over time, reaching a mean estimated score of 52.5 close to 2 years post-diagnosis. Despite over half of the sample having PCS scores above population norms, 42% of women were in a Low Group with a very low estimated mean PCS of 36.4 soon after diagnosis, which did not improve over time.

### Characteristics of PCS trajectory group membership

Bivariate characteristics associated with trajectory group membership are shown in Table 2. Women in the Low Group, compared to those in the High Group, were significantly more likely to be older (M = 74.1 years versus 71.4 years, *p* = 0.009), report having difficulty paying for basics (23% versus 4%, *p* = 0.003), have a greater number of comorbidities (*p* = 0.005), and higher BMI (M = 28.9 versus 24.9, *p* = 0.0002). The Low Group also had significantly higher estimated illness intrusiveness means at four months post-diagnosis (M = 21.9 vs 16.9, *p* = 0.005), lower social support (M = 3.9 versus 4.3, *p* = 0.001), lower optimism (M = 21.1 versus 23.2, *p* = 0.02), and lower meaning and peace (M = 22.9 versus 25.8, *p* = 0.005). Cancer-related characteristics (cancer stage and treatment), race/ethnicity, marital status, and coping styles did not differ significantly between groups. Time since diagnosis also did not vary significantly between the two groups.

**Table 2:** Characteristics associated with PCS trajectory group membership in bivariate analyses

	Trajectory Group		
	Low (*N* = 61)	High (*N* = 84)	
Characteristics	n (%) or Mean (*SE*)	n (%) or Mean (*SE*)	*p*
*Sociodemographic Variables*			
Age at diagnosis, years	74.1 (0.9)	71.4 (0.6)	**0.009**
Time since diagnosis, months	4.6 (0.2)	4.8 (0.1)	0.20
Non-Hispanic Caucasian	56 (92)	81 (96)	0.3
College graduate	23 (38)	45 (54)	0.06
Married/partnered	36 (59)	57 (68)	0.3
Difficulty to pay for basics, somewhat/very hard	14 (23)	3 (4)	**0.0005**
*Cancer-related Variables*			
Cancer Stage			0.2
I	36 (59)	58 (69)	
II-III	25 (41)	26 (31)	
Mastectomy, yes^a^	16 (26)	20 (24)	0.7
Radiation, yes^a^	41 (67)	61 (73)	0.5
Chemotherapy, yes^a^	23 (38)	32 (38)	> 0.9
Hormonal therapy	49 (80)	68 (81)	> 0.9
*Health-related Variables*			
BMI, kg/m2	28.9 (0.9)	24.9 (0.5)	** < 0.0001**
Comorbidities, No			**0.0005**
0	1 ( 2)	13 (16)	
1	11 (18)	25 (30)	
2	14 (23)	23 (28)	
3 +	35 (57)	22 (27)	
*Psychosocial Variables *^*b*^			
Illness intrusiveness	21.9 (1.3)	16.9 (1.2)	**0.005**
Coping			
Active coping	2.5 (0.1)	2.5 (0.1)	> 0.9
Passive coping	1.3 (0.05)	1.3 (0.04)	0.3
Social Support	3.9 (0.1)	4.3 (0.1)	**0.001**
Spirituality			
Meaning & Peace	22.9 (0.8)	25.8 (0.7)	**0.005**
Role of Faith	9.6 (0.6)	10.9 (0.5)	0.1
Optimism	21.1 (0.6)	23.2 (0.6)	**0.02**

*Abbreviations*: BMI, Body Mass Index; PCS, Physical Component Summary

^a^At any time following diagnosis

^b^Values are estimated at 4-months post diagnosis

*p*-values < 0.05 are bolded

Predictors significant at *p* ≤ 0.1 from the bivariate analyses were included in a multivariable logistic regression model. Age, comorbidities, BMI, and psychosocial variables were entered as continuous variables, so that each reported odds ratio (OR) for these variables represents the OR associated with a 1-unit increase in the value of the predictor. Older age in years (OR = 1.09, 95% CI = 1.01–1.18,* p* = 0.02), difficulty paying for basics (OR = 6.10, 95% CI = 1.29–28.88,* p* = 0.02), higher level of comorbidity category (OR = 1.85, 95% CI = 1.15–2.96, *p* = 0.01), and higher BMI (OR = 1.12, 95% CI = 1.02–1.23, *p* = 0.02) were significant predictors of PCS Low Group membership (Table 3). Psychosocial variables were no longer significant in the multivariable setting. We investigated whether multicollinearity in the logistic model explained why none of the psychosocial variables was significantly associated with trajectory group membership. Intercept-adjusted condition indices were in the modest range (range from 1.00 to 3.04; see supplemental Table 1); thus, it is possible that a mild degree of collinearity among the psychosocial variables affected model results, but the collinearity did not appear strong enough to require changes to the model in terms of dropping any variables.

**Table 3:** Multivariable logistic regression predicting low PCS trajectory group membership

Variables	Odds Ratio	95% Confidence Intervals	*p* value
Age at diagnosis, years	1.09	1.01–1.18	**0.02**
College graduate	1.20	0.46–3.12	0.7
Difficulty to pay for basics, somewhat/very hard	6.10	1.29–28.88	**0.02**
BMI, kg/m2	1.12	1.02–1.23	**0.02**
Comorbidities, No	1.85	1.15–2.96	**0.01**
Illness intrusiveness	1.05	1.00–1.11	0.07
Social Support	0.62	0.34–1.13	0.1
Spirituality, Role of Faith	0.93	0.83–1.04	0.2
Spirituality, Meaning & Peace	0.98	0.87–1.10	0.7
Optimism	1.04	0.93–1.16	0.5

*Abbreviations:* BMI, Body Mass Index; PCS, Physical Component Summary

*p*-values < 0.05 are bolded

Given that number of medical comorbidities was a strong predictor of PCS group membership, we conducted an exploratory bivariate analysis to examine specific comorbidities that distinguished between trajectory groups. These included arthritis, hypertension, as well as other comorbidities categorized by organ system dysfunction [45]: cardiovascular disease (angina, heart disease), gastrointestinal disease (Crohn’s disease, diverticulitis, gallbladder disease, pancreatitis, ulcers), respiratory disease (asthma, emphysema), and thyroid disease (hyperthyroidism, hypothyroidism). Women in the Low Group, compared to the High Group, were more likely to have arthritis (66% versus 30%, *p* < 0.001), hypertension (62% versus 46%, *p* = 0.05), cardiovascular disease (23% versus 10%, *p* = 0.03), gastrointestinal disease (23% versus 10%, *p* = 0.03), and respiratory disease (18% versus 6%,* p* = 0.02).

## Discussion

This study examined physical HRQoL trajectories in women aged ≥ 65 years diagnosed with breast cancer. We found two distinct PCS trajectories in our sample. One group, constituting 58% of our sample, had high PCS following diagnosis that improved slightly over time, and the second group (42%) had consistently low PCS that did not improve over time. In a multivariable model, older age, difficulty paying for basics, higher BMI, and greater number of medical comorbidities were predictive of low PCS group membership. Cancer-related and treatment-related factors were not significant predictors, nor were any of the psychosocial variables still significantly associated with trajectory group in the multivariable setting.

In the first 2 years after a breast cancer diagnosis, the majority of survivors in our sample had similar or better physical HRQoL than population reference norms of women ≥ age 65 without a history of cancer [7, 8]. This is an important finding, as cross-sectional and longitudinal studies that do not consider heterogeneity among older breast cancer survivors women suggest that older survivors in general, report significantly lower physical HRQoL compared to age-matched adults without a history of cancer [11–13].

Nonetheless, 42% of women were in a low PCS group that did not improve over time. In fact, survivors in this group scored six to nine points lower on PCS compared to population reference norms of older women without a history of cancer [7, 8], exceeding the minimally important difference of two to three points on the PCS [11, 46]. In contrast to other studies of HRQoL trajectories [29, 31, 32], we did not find a group that declined over time. It is possible that a longer follow-up time would show greater heterogeneity and a declining group [31].

Consistent with previous research [15, 29, 30, 32, 47], we found several sociodemographic, health-related, and psychosocial variables that significantly distinguished between trajectory groups in bivariate analyses. Cancer-related variables (e.g. cancer stage, treatment) did not significantly predict group membership [29, 31]. In the multivariable analysis, older age, difficulty paying for basics, higher BMI, and greater number of comorbidities were significantly associated with the low PCS trajectory group [15, 29, 32, 47].

It is also worth noting that although a small percentage of survivors reported difficulty paying for basics, women in the low PCS group had six times the odds of having a somewhat/very hard ability to pay for basics compared to the high PCS group. This finding is consistent with the literature on associations between low socioeconomic status and poor physical HRQoL in breast cancer survivors [48–51]. Incorporating screenings such as the Comprehensive Score for Financial Toxicity-Functional Assessment of Chronic Illness Therapy [52] may be an efficient way to identify vulnerable patients at risk for financial toxicity [53]. This may result in earlier referrals for social workers, financial counselors, transportation vouchers, and co-pay assistance resources to decrease financial toxicity [54].

BMI was another significant predictor. Women in the low PCS group were significantly more likely to be overweight compared to the high PCS group, which averaged in the normal BMI range. This finding is consistent with the literature on the relationship between obesity and physical health [55]. Obesity is a risk factor for chronic health conditions including breast cancer, and is associated with greater treatment complications, less effective treatment results, likelihood for recurrence and increased mortality [55, 56], all of which may contribute to poorer physical HRQoL.

The finding that number of comorbidities was a highly significant predictor of PCS is consistent with other studies [31, 32]. More than half (57%) of cancer survivors in the low PCS group reported having at least *three* medical comorbidities, with two-thirds of women in this group having arthritis or hypertension. In fact, survivors in the low PCS group were more than twice as likely to have arthritis, cardiovascular, gastrointestinal, or respiratory disease, all of which negatively impact physical HRQoL [57–59]. Given that presence of comorbidities may limit treatment options, increase risk of toxicity, and negatively impact survivorship [60], identification of specific comorbidities and/or clusters may help predict poorer PCS trajectories in older women with breast cancer. The National Comprehensive Cancer Network recommends use of comorbidity assessment tools to older patients prior to cancer treatment [61]. However, a systematic review on the utilization of comorbidity tools suggest limited use in clinical practice [62]. Barriers to implementation may be due to time constraints and lack of perceived value or benefit from clinicians [62]. Nonetheless, our finding that number of medical comorbidities was a primary variable associated with poorer PCS trajectories suggests the importance of these in geriatric oncology care. Assessing the presence or absence of comorbidities using tools such as the Charlson Comorbidity Index (CCI) or Cumulative Illness Rating Scale-Geriatric (CIRS-G) may facilitate treatment decision making, determine toxicity risk, identify vulnerable patients, and enhance treatment success. Potential interventions may include optimizing specific health conditions prior to cancer treatment and coordinating a multidisciplinary treatment team consisting of oncologists, primary care physicians, psychologists, and other specialists to optimize health and well-being [61, 63].

The incorporation of routine comprehensive geriatric assessments (CGA) that screen for the above factors may help providers identify older women at risk for poorer physical HRQoL post breast cancer treatment. The International Society of Geriatric Oncology (SIOG) guidelines recommend clinicians to routinely administer CGA as an effective evidence-based care model for older adults with cancer to assist with assessment and discussion of treatment options [64, 65]. These assessments typically include the following domains: functionality, nutrition, cognition, psychological state, social support, comorbidities, medications, and geriatric syndromes [65]. A recent systematic review of utilization of CGA in older women with early stage non-metastatic breast cancer suggested that results of the CGA can be successfully used to assess QOL and predict treatment outcomes (e.g. survival and mortality rate) [66]. As such, CGA may assist with identifying varying levels of fitness of patients based on an algorithm of factors to determine the most optimal treatment options that increase chances of survival, minimize treatment adverse effects, and decrease risk for poorer physical HrQOL outcomes [66]. Although there is no current gold standard for CGA, our results suggest that age, number of comorbidities, BMI, and financial hardship may be important domains to consider. Future research is encouraged to explore the domains of the CGA that are most applicable for older women diagnosed with breast cancer to assist with cancer treatment options and treatment decision making to optimize survival and health outcomes.

There are several limitations to this study. A larger sample or longer follow-up may have identified a greater number of trajectories, and/or allowed for a more thorough examination of the impact of specific medical comorbidities on PCS group membership. Baseline assessment occurred between 3 days and 8 months following diagnosis, and thus our reported mean values of the psychosocial covariates at 4 months post-diagnosis are estimates, specifically least-squares estimates derived from repeated measures models. Age and gender-based population reference norms were used to compare the PCS scores to older women without a history of cancer. Our sample was racially and socioeconomically fairly homogeneous, which limits generalizability. Finally, we recognize it is unlikely that the MCAR assumption underlying trajectory analysis hold fully in these or any observational data; this is an additional reason for caution in generalizing our findings.

## Conclusion

This paper contributes to the geriatric oncology literature by examining the PCS trajectories among older cancer survivors in the early years post-breast cancer diagnosis. Although the majority of survivors reported high PCS, a large subgroup reported consistently low PCS that did not improve over time. Older age, BMI, ability to pay for basics, and number of medical comorbidities were strongly associated with low PCS group membership. Incorporating comprehensive geriatric assessments that assess for these characteristics may help identify individuals at risk for poorer physical HRQoL post breast cancer treatment.

## Data Availability

Data sets generated during the current study are available from the corresponding author on reasonable request.

## Abbreviations

BMI: Body mass index
HRQoL: Health-related quality of life
PCS: Physical component summary score
SF-36: Medical health outcomes short-form survey
CGA: Comprehensive Geriatric Assessment
